# Quality of life in *SCN1A*-related seizure disorders across the lifespan

**DOI:** 10.1093/braincomms/fcae285

**Published:** 2024-08-26

**Authors:** Crista A Minderhoud, Amber Postma, Floor E Jansen, Janneke R Zinkstok, Judith S Verhoeven, Bianca Berghuis, Wim M Otte, Marian J Jongmans, Kees P J Braun, Eva H Brilstra

**Affiliations:** Department of Child Neurology, UMCU Brain Center, University Medical Center Utrecht, Utrecht, The Netherlands; Department of Psychiatry, UMCU Brain Center, University Medical Center Utrecht, 3583CX Utrecht, The Netherlands; Department of Child Neurology, UMCU Brain Center, University Medical Center Utrecht, Utrecht, The Netherlands; Department of Psychiatry, UMCU Brain Center, University Medical Center Utrecht, 3583CX Utrecht, The Netherlands; Department of Psychiatry, Radboud University Medical Center, 6525GA Nijmegen, The Netherlands; Karakter Child- and Adolescent Psychiatry, 6525GC Nijmegen, The Netherlands; Department of Child Neurology, Academic Centre for Epileptology Kempenhaeghe, 5590AB Heeze, The Netherlands; Stichting Epilepsie Instellingen Nederland, 8025BV Zwolle, The Netherlands; Department of Child Neurology, UMCU Brain Center, University Medical Center Utrecht, Utrecht, The Netherlands; Department of Pedagogical and Educational Sciences, Faculty of Social and Behavioral Sciences, Utrecht University, 3584CS Utrecht, The Netherlands; Department of Neonatology, Wilhelmina Children’s Hospital, University Medical Center Utrecht, 3584EA Utrecht, The Netherlands; Department of Child Neurology, UMCU Brain Center, University Medical Center Utrecht, Utrecht, The Netherlands; Department of Genetics, UMC Utrecht Brain Center, University Medical Center Utrecht, 3583CX Utrecht, The Netherlands

**Keywords:** HRQoL, Dravet syndrome, epilepsy, SCN1A, behaviour

## Abstract

This cohort study aims to describe the evolution of disease features and health-related quality of life per life stage in Dravet syndrome and other *SCN1A*-related non-Dravet seizure disorders which will enable treating physicians to provide tailored care. Health-related quality of life and disease features were assessed cross-sectionally in participants with a *SCN1A-*related seizure disorder, categorized per age group for Dravet syndrome, and longitudinally over seven years follow-up (2015–2022). Data were collected from questionnaires, medical records, and semi-structured telephonic interviews. Health-related quality of life was measured with the Paediatric Quality of Life Inventory, proxy-reported for participants with Dravet syndrome and for participants with non-Dravet aged younger than 18 years old and self-reported for participants with non-Dravet over 18 years old. Associations between health-related quality of life and disease features were explored with multivariable regression analyses, cross-sectionally in a cohort of 115 patients with Dravet and 48 patients with generalized epilepsy with febrile seizures plus and febrile seizures (non-Dravet) and longitudinally in a cohort of 52 Dravet patients and 13 non-Dravet patients. In the cross-sectional assessment in 2022, health-related quality of life was significantly lower in Dravet syndrome, compared to non-Dravet and normative controls. Health-related quality of life in the School and Psychosocial domain was significantly higher in older Dravet age groups. A higher health-related quality of life was associated with fewer behavioural problems [*β* = −1.1; 95% confidence interval (CI), (−1.4 to −0.8)], independent walking (*β* = 8.5; 95%CI (4.2–12.8)), compared to the use of a wheelchair), and fewer symptoms of autonomic dysfunction (*β* = −2.1, 95%CI (−3.2 to −1.0)). Longitudinally, health-related quality of life was significantly higher seven years later in the course of disease in Dravet participants (Δ8.9 standard deviation (SD) 18.0, *P* < 0.05), mediated by a lower prevalence of behavioural problems (*β* = −1.2, 95%CI (−2.0 to −0.4)), lower seizure frequency (*β* = −0.1, 95%CI (−0.2 to −0.0)) and older age (*β* = 0.03, 95%CI (0.01–0.04)). In summary, health-related quality of life was significantly higher at older age in Dravet syndrome. This finding may reflect the benefits of an advanced care strategy in recent years and a ceiling of severity of disease symptoms, possibly resulting in an increased wellbeing of parents and patients. The strong association with behavioural problems reinforces the need to incorporate a multidisciplinary approach, tailored to the age-specific needs of this patient group, into standard care.

## Teaser

Minderhoud *et al.* report that health-related quality of life significantly improves with age in *SCN1A*-related Dravet syndrome and is associated with less behavioural problems and a lower seizure frequency. Based on age-specific data, they delineated a distinct Dravet disease profile per life stage to function as a blueprint for tailored treatment.

## Graphical Abstract

**Graphical abstract:**
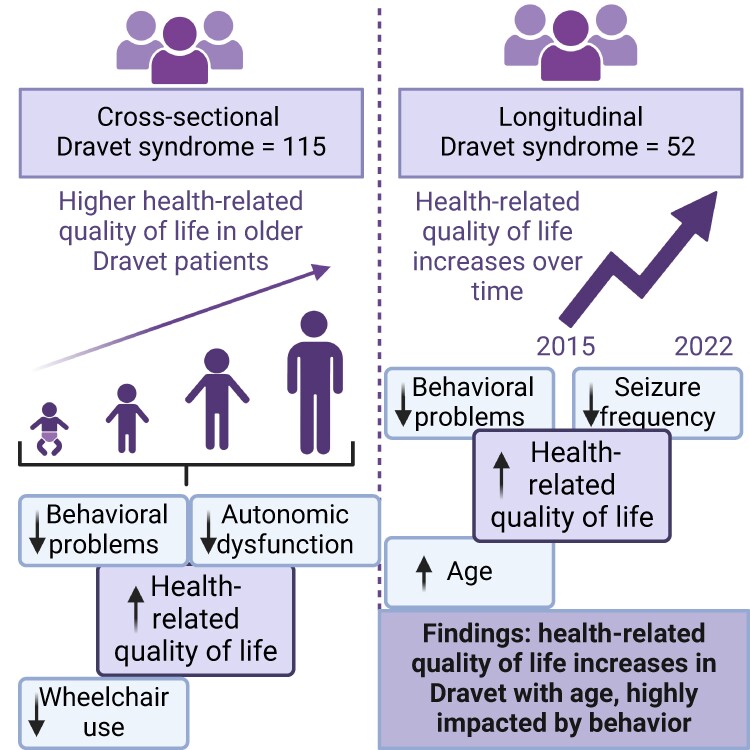

## Introduction

*SCN1A*-related seizure disorders are caused by pathogenic variants in the *SCN1A*-gene—which codes for the alpha subunit of a voltage-gated sodium channel and is predominantly expressed in GABAergic inhibitory interneurons—leading to increased neuronal excitability.^1^ These disorders vary greatly in severity, with the milder end of the spectrum non-Dravet syndrome (non-DS) phenotypes, such as genetic epilepsy with febrile seizures plus (GEFS+) and febrile seizures (FS) with neurodevelopmental outcome in the normal range.^2,3^ Dravet syndrome (DS), on the severe end of the spectrum, is a developmental and epileptic encephalopathy (DEE), presenting in the first year of life with prolonged generalized or unilateral tonic-clonic or clonic, fever-sensitive seizures. After the first year of life, patients develop often therapy-resistant epilepsy, with multiple seizure types, e.g. atypical absences and myoclonic seizures,^4^ and neurodevelopment slows, resulting in intellectual disability (ID) and functional motor impairment. Behavioural problems, such as autistic traits and Attention Deficit Hyperactivity Disorder (ADHD)-like features, often occur.^4,5^

The disease evolves across the lifespan of DS patients, with some disease features emerging as more burdensome, while others dampen.^6,7^ Over recent years, there has been a growing recognition of relevant comorbidities, such as sleep disturbances, gastrointestinal and eating problems and autonomic dysfunction, that highly impact quality of life.^8-12^ Previous research has established that patients with *SCN1A*-related DS experience lower health-related quality of life (HRQoL) than patients with another chronic illness and the general population.^13-15^ Behavioural problems, as well as a higher seizure frequency, have been reported to be strongly predictive of poorer HRQoL.^13,14,16,17^ However, prospective data that provides a comprehensive overview of HRQoL and its relation with other evolving disease features is scarce.

With this study, we aimed to describe the evolution of the clinical characteristics and HRQoL of *SCN1A*-related seizure disorders over time, by a cross-sectional exploration and analysis of disease features and HRQoL per age group, and by a follow-up of HRQoL and disease features within individual patients measured at two timepoints 7 years apart. Determining which disease features are most relevant per life stage and their impact on HRQoL will enable tailored, more effective care and counselling, to improve quality of life for patients and caregivers.

## Materials and methods

### Study design and population

The current study is a follow-up study of a cohort of patients with *SCN1A-*related seizure disorders described in 2015,^13,18^ which we expanded with additional participants. Consequently, this study has a dual study structure. We performed a longitudinal cohort study over 7-year follow-up (2015–2022) and a cross-sectional observational cohort study (2022). Participants from the 2015 cohort who had consented to be contacted for participation in a follow-up study were approached for inclusion in the current study. These participants were eligible for both the longitudinal and cross-sectional cohort. The cross-sectional cohort was expanded with patients who were diagnosed with a *SCN1A-*related seizure disorder after 2015 or who did not participate in 2015. They were invited to participate by their physician, or responded to an invitation from the parent/patient organization ‘Dravet syndrome Foundation Netherlands/Flanders’.^19^ A flowchart (Supplementary Fig. 1) illustrates how the cohort is constructed.

Participants were eligible for inclusion if they were diagnosed with Dravet syndrome, GEFS + or FS and carried a heterozygous pathogenic, or likely pathogenic variant in *SCN1A* (classes IV and V, according to the American College of Medical Genetics and Genomics criteria).^20^ The diagnostic categorization between DS and non-DS phenotypes was made by the treating physician and verified by the researchers with the most recent guidelines.^21^ Patients <2 years old were excluded, because most questionnaires are not validated in this age group.

This study is part of a larger research project, on quality of life and behaviour in *SCN1A*-related seizure disorders. Part of this cohort has been included in recent publications.^12,22^ Data collection at the first timepoint, in 2015, has been described previously.^13,18^ Data collection at the second timepoint took place between October 2021 and December 2022. We used the STROBE cohort checklist when writing our report.^23^

### Standard protocol approvals and patient consent

The local Medical Research Ethics Committee (MREC) Nedmec approved the study design (21–561). Informed consent was obtained from participants before inclusion in the study, according to the Declaration of Helsinki. For minors or incapacitated subjects, informed consent was given by their parents or legal guardians.

### Data collection

At both timepoints, clinical data on genetic diagnosis, epilepsy characteristics, development, comorbidities and medication use were collected retrospectively from medical records and age-specific questionnaires filled in by participants or parents of participants. The flowchart in Supplementary Fig. 1 illustrates which data was collected per timepoint. In the longitudinal study, only participants for whom completed HRQoL data were available at both timepoints were included. Therefore, participants for whom no HRQoL data was available from 2015 were included in the cross-sectional study only. Inherent to their level of ID, all questionnaires administered to DS participants were proxy-reported and completed by their parents. For non-DS participants aged younger than 18 years old, all questionnaires were proxy-reported, by parents. For non-DS participants over 18 years old, all questionnaires were self-reported. Proxy- and self-reported scores were analysed separately.

### Outcome measure

The outcome measure, HRQoL, was assessed with the Paediatric Quality of Life Inventory (PedsQl),^24^ which ranks HRQoL on a scale of 0–100, with higher scores indicating higher HRQoL. Twenty-five questions are answered on a five-point Likert scale (0 = ‘never a problem’ to 4 = ‘almost always a problem’). The measure consists of five scales: Total score, which is the primary outcome measure of this study, and the scores addressing the Physical, Emotional, Social and School domains. The Psychosocial domain is an additional scale computed from the latter three scores. In the 2022 non-DS group, adult participants completed the self-report questionnaire. In 2015, only proxy-reported PedsQl questionnaires were obtained. Therefore, there are no longitudinal self-report scores available.

### Disease characteristics

Intellectual disability (ID) was rated on a five-point Likert scale based on intelligence quotient (IQ), and developmental age, from which IQ can be approximated [no ID (IQ > 85), borderline ID (IQ 70–85), mild ID (IQ 50–70), moderate ID (IQ 30–50) or severe to profound ID (IQ < 30)]. This categorization is largely based on the DSM IV classification for intellectual disability,^25^ but minimally deviates in the distinction between severe and moderate ID (a cut-off IQ-value of 30 versus 35), in order to conform to the 2015 categorization. When no recent IQ or DQ was available (*n* = 39), i.e. no formal assessment was performed in the prior 3 years, ID was categorized, by consensus of a child neurologist, neuropsychologist and clinical geneticist, based on past IQ or development assessments, received education or day-care, communication skills and daily functioning.

Epilepsy severity was rated with several measures. (i) Seizure frequency during data collection was categorized as seizure freedom (>1 year), yearly, monthly, weekly or daily seizures. (ii) Seizure types were categorized as minor (absences, focal seizures with intact awareness and myoclonus) or major (generalized tonic-clonic, hemiclonic, tonic, atonic and focal seizures with impaired awareness). (iii) Overall epilepsy severity was rated with the Early Childhood Epilepsy Severity Scale (E-Chess),^26^ ranking five indicators on a scale of 1–3. The higher the score, the more severe the epilepsy.

Functional mobility was rated with the Dutch version of the Functional Mobility Scale (FMS) questionnaire, validated in participants >4 years old, which reflects functional mobility on three distances (5, 50 and 500m).^27^ We reported the outcome for 500 meters. In all analyses, the FMS score was simplified into a dichotomous variable (wheelchair versus independent walking).

Behavioural problems were assessed with the Dutch parent-report version of the Child Behaviour Checklist 1.5–5 (CBCL), Child Behaviour Checklist 6–18, the Adult Behaviour Checklist 18–59 (ABCL) or the Adult Self-Report 18–59 (ASR). In the non-DS group, adult participants completed the self-report questionnaire. *T*-scores that compare the scores to norm groups from the general population were calculated. Based on *T*-scores of the total problems scale, participants were divided into normal, borderline and clinical range behavioural problems.^28,29^

Sleep problems were quantified with the Dutch translation of the Sleep Behaviour Questionnaire by Simonds & Parraga (SQ-SP), modified for use in individuals with ID.^30,31^ For this study, we calculated the Composite Sleep Index (CSI), which ranges 0–12. A score of four or higher indicates a severe sleeping problem.

Gastrointestinal and eating problems were measured with a questionnaire, developed by the research team, with a dietitian and two speech therapists (Supplementary questionnaire 1). This questionnaire assessed the presence and frequency of nine gastrointestinal or eating symptoms, discussed in detail in previous work.^12^ For this study, these symptoms were reported as ‘number of gastrointestinal and eating problems present ≥ weekly’. Autonomic dysfunction was evaluated with a questionnaire (Supplementary questionnaire 2). The presence of ten symptoms of autonomic dysfunction for at least 10 minutes was rated on a frequency scale, only if they occurred interictally and without an environmental trigger. For this study, these symptoms were reported as ‘number of symptoms of autonomic dysfunction present ≥ weekly’.

The overall impact of the disease was assessed in a telephonic interview with a composite impact score, based on how many aspects of life (family life, siblings and relationship of parents) are negatively influenced by the disease features.

### Statistical analyses

To explore potential selection bias, differences between non-responders and responders from the 2015 cohort were analysed with a two-sample *t*-test for continuous variables and a Fisher's Exact test for categorical variables.

For the 2022 cross-sectional cohort, baseline characteristics and HRQoL scores are given for (1) DS proxy-reported, (2) non-DS proxy-reported and (3) non-DS self-reported. The DS cohort was divided into four age groups: 2–6 years old, 7–12 years old, 13–20 years old and >20 years old. Differences in HRQoL and prevalence of disease features between the age groups were assessed with an ANOVA and a post-hoc Tukey´s Honestly Significant Difference (HSD) for continuous variables, and a chi-square test or Fisher's exact test, for categorical variables. Missing data were imputed by creating 50 different datasets with imputed data with the Multiple Imputation by Chained Equations (MICE) method.^32^ First, a majority selection method was performed on each imputed dataset. Variables that were present in at least half of the models were eligible for selection in the final model. Then, the final model was created using stepwise model selection based on the Wald statistic calculated from the imputed data. For the final model, the imputed data were pooled into single estimates with Rubin's rule.^33^

Multiple linear regression analyses were performed, first with the primary outcome variable HRQoL total score, and subsequently the HRQoL domain scores separately, for (1) DS proxy-reported, (2) non-DS proxy-reported and (3) non-DS self-reported, and (4) proxy-reported per age group of DS participants. Associations with the continuous variables behavioural problems (total problems *T*-score), sleeping difficulties (CSI score), age and the ordinal variables major seizure frequency, ID level, functional mobility, gastrointestinal and eating problems, autonomic dysfunction, the composite impact score and the categorical variable sex were analysed. In the ID level variable, the categories no ID and borderline ID were merged into one category.

The FMS is not validated in the age group 2–4 years old; therefore, a sensitivity analysis excluding this age group was performed.

For the longitudinal cohort, descriptives are depicted for participants for whom HRQoL scores are available at both timepoints. For the non-DS group, data are shown for the group who completed proxy-reported questionnaires at both timepoints, since no self-reported questionnaires were administered in 2015 and proxy- and self-reported questionnaires cannot be pooled and compared. To assess whether HRQoL scores and disease features significantly differed between baseline and follow-up, a paired *t*-test or Wilcoxon signed rank test for continuous variables or Fisher's Exact Test for categorical variables was performed. Data were imputed according to the same procedure as in the cross-sectional analysis. A multiple linear regression analysis was carried out with the percentage change in HRQoL total score between 2015 and 2022 as the outcome, computed by dividing the absolute change in HRQoL between timepoints by the HRQoL at the first timepoint. Associations were tested with the percentage change in the continuous variable behavioural problems and the absolute change in the ordinal variables major seizure frequency, ID level and functional mobility. Age, sex and the composite impact score in 2022 were also included in the analysis.

For all models, an *R*^2^ was calculated to represent the proportion of variance for the outcome explained by the model. Collinearity was checked by computing the Variance Inflation Factors. All tests were performed two-tailed with an alpha-level of significance of *P* < 0.05. Statistical analyses were performed using R-studio (version 1.4.1106). Figures were created in Graphpad Prism (version 10.0.3) and Biorender.

## Results

### HRQoL in the 2022 cross-sectional cohort

The 2022 cross-sectional cohort consisted of 163 participants, of whom 115 participants with DS (70.6%), 21 proxy-reported non-DS participants (12.9%) and 27 self-reported non-DS participants (16.6%). The cohort included 101 participants of the original 2015 cohort, and 62 participants who were diagnosed or identified after 2015. When comparing the 101 responders to the 75 non-responders from the 2015 DS cohort (*n* = 176), non-responders are significantly older and less mobile (Supplementary Table 1). There was a mortality of 15.8% amongst DS participants. In the non-DS cohort, non-responders have a significantly higher ABCL/CBCL total problems *T*-score, but lower than the borderline or clinical range.

**Figure 1:**
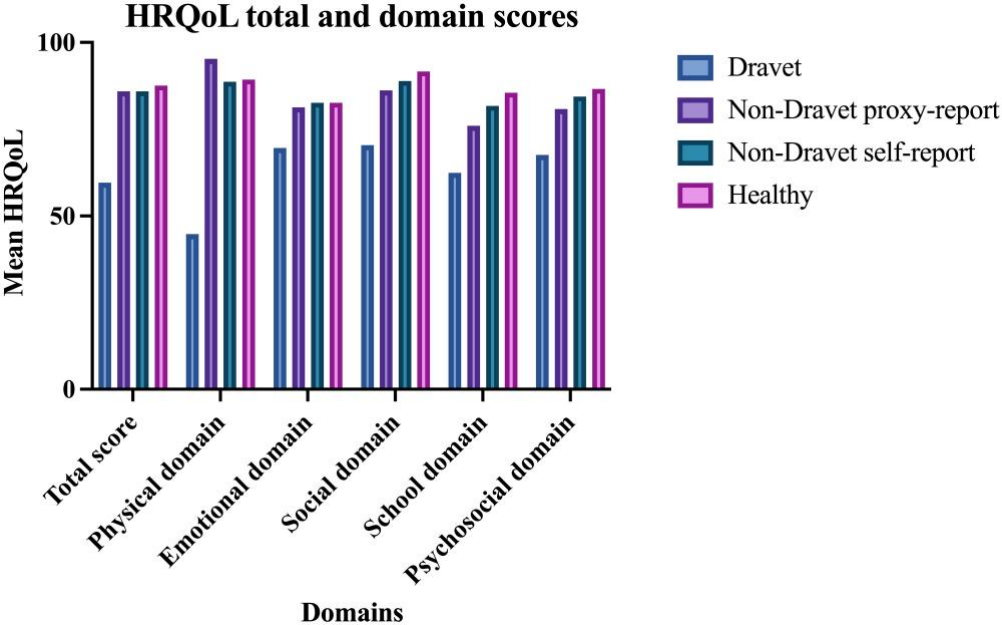
**HRQoL total and domain scores for the total cohort, and normative controls.** Normative data for the healthy control group are proxy-reported.^34^ Group differences for all total and domain scores were tested with an ANOVA and a post-hoc Tukey's HSD. The difference between DS and the other three groups was significant for all scores; the total HRQoL score (F-value 158.2, *P* < 0.0001), the Physical domain score (*F-*value 215.3, *P* < 0.0001), the Emotional domain score (*F*-value 18.7, *P* < 0.05), the Social domain score (*F*-value 59.6, *P* < 0.001), the School domain score (*F*-value 47.0, *P* < 0.05) and the Psychosocial domain score (*F*-value 136.2, *P* < 0.0001). Differences between the non-DS groups and the normative control group were not significant. DS = Dravet syndrome non-DS = non-Dravet phenotypes HRQoL = health-related quality of life. Figure created with Graphpad Prism.

Table 1 provides an overview of the demographic and clinical characteristics. In Fig. 1. HRQoL total and domain scores for DS and non-DS are depicted, along with normative data of healthy proxy-reported controls for reference.^34^ The mean total score for HRQoL in DS participants was 59.6. DS participants score lowest on the Physical domain (44.8), and highest on the Social domain (70.4). The scores for the non-DS group are considerably higher, with a mean total score of 85.9 for both the proxy-reported and self-reported scores. For the HRQoL total score and all domain scores, the difference between the DS group and the other three groups was statistically significant. Differences between the non-DS groups and the healthy control group were not significant.

**Table 1:** Characteristics of the 2022 cross-sectional study population

	Dravet	Non-Dravet proxy-reported	Non-Dravet self-reported
*N* (%)	115 (70.6)	21 (12.9)	27 (16.6)
Sex: female (%)	57/115 (49.6)	15/21 (71.4)	16/27 (59.3)
Mean age in years (SD)	17.2 (11.2)	11.3 (3.1)	39.1 (15.8)
- *Missing*	0	0	0
Developmental level *n* (%)^a^			
- No to borderline ID	7/115 (6.1)	21/21 (100.0)	27/27 (100.0)
- Mild ID	37/115 (32.2)	0	0
- Moderate ID	28/115 (24.3)	0	0
- Severe ID	43/115 (37.4)	0	0
			
Mean total e-chess score (SD)	12.9 (3.2)	7.1 (2.1)	6.6 (3.0)
- *Missing*	2	2	5
Minor seizure frequency *n* (%)			
- No minor seizures	40/115 (34.8)	17/21 (81.0)	25/27 (92.6)
- Yearly	10/115 (8.7)	3/21 (14.3)	1/27 (3.7)
- Monthly	11/115 (9.6)	1/21 (4.8)	0
- Weekly	17/115 (14.8)	0	1/27 (3.7)
- Daily	37/115 (32.2)	0	0
Major seizure frequency, *n* (%)			
- No major seizures	9/115 (7.8)	10/21 (47.6)	18/27 (66.7)
- Yearly	26/115 (22.6)	10/21 (47.6)	8/27 (29.6)
- Monthly	29/115 (25.2)	1/21 (4.8)	1/27 (3.7)
- Weekly	39/115 (33.9)	0/21	0/27
- Daily	12/115 (10.4)	0/21	0/27
Current use of anti-seizure medication, *n* (%)			
- No ASM	1/114 (0.9)	11/21 (52.4)	13/26 (50.0)
- 1–2 ASM	27/114 (23.7)	10/21 (47.6)	11/26 (42.3)
- ≥3 ASM	86/114 (75.5)	0	2/26 (7.7)
Use of CIM, *n* (%)	72/114 (63.2)	4/19 (20.0)	12/26 (46.2)
Use of ketogenic diet, *n* (%)			
- In the past	23/114 (20.2)	0/21	1/26 (3.8)
- Currently	2/114 (1.8)	0	0
Use of vagal nerve stimulator, *n* (%)			
- In the past	5/114 (4.4)	0/21	1/26 (3.7)
- Currently	7/114 (6.1)	0	0
			
Functional mobility score 500meters, *n* (%)^b^			
- Uses a wheelchair (1) or a walker (2)^c^	48/108 (44.4)	0	1/26 (3.8)
- Independent walking on flat surfaces (5)	31/108 (28.7)	0	0
- Independent walking on all surfaces (6)	29/108 (26.9)	44/19 (97.8)	44/26 (97.8)
ABCL/CBCL/ASR total behavioural problems score, *n* (%)^d^			
- Normal range	37/104 (35.6)	12/17 (70.6)	17/22 (77.3)
- Borderline range	22/104 (21.2)	2/17 (11.8)	5/22 (22.7)
- Clinical range	45/104 (43.3)	3/17 (17.6)	0
			
Signs of autonomic dysfunction, *n* (%)			
- 3 or more symptoms on a weekly basis	37/113 (32.7)	1/21 (4.8)	3/27 (11.1)
Gastrointestinal and eating problems, *n* (%)			
- 3 or more symptoms on a weekly basis	41/115 (35.7)	0/21	0/27
Sleeping problems^e^			
CSI > 4 *n* (%)	21/115 (18.3)	1/19 (5.3)	NA

Prevalence is depicted as occurrence/total (percentage). For continuous variables, missings are displayed separately.

ASM = anti-seizure medication, CIM = contra-indicated medication, CSI = composite sleeping index as measured by the SPSQ ID = intellectual disability SD = standard deviation.

^a^No intellectual disability (ID) = IQ > 85, borderline ID = IQ 70–85, mild ID = IQ 50–70, moderate ID = IQ 30–50, severe to profound ID = IQ < 30.

^b^The outcome for 500 meters distance on the Dutch version of the Functional Mobility Scale (FMS).

^c^Only one non-DS participant used a walker, all DS participants used a wheelchair.

^d^Based on the Dutch parent-report version of the Child Behaviour Checklist 1.5–5 years (CBCL), Child Behaviour Checklist 6–18 years, the Adult Behaviour Checklist 18–59 years (ABCL), or the Adult Self-Report 18–59 (ASR).

^e^Based on the Dutch translation of the Sleep Behaviour Questionnaire by Simonds & Parraga (SQ-SP), modified version for use in individuals with intellectual disability. The CSI was not computed for the adult questionnaire.

Table 2 shows the results of the multivariable regression analysis for the HRQoL total and domain scores for DS and the total HRQoL score for non-DS self-report. In DS participants, more severe behavioural problems and more signs of dysautonomia were significantly associated with lower total HRQoL. Independent mobility on 500 m was significantly associated with a higher total HRQoL. When performing a sensitivity analysis excluding participants <4 years old, the impact score was no longer of added value in the Emotional domain score, other analyses yielded similar results. In the non-DS proxy-reported participants, there was no significant association between HRQoL and the disease features. For the non-DS group who self-reported, a younger age and less behavioural problems were associated with a higher HRQoL total score.

**Table 2:** Results from the multivariable regression analyses for the cross-sectional cohort with HRQoL total score, depicted for DS and non-DS self-report, and HRQoL domain scores, for DS, as the outcome measure

		Outcome measures						
		Non-DS self-report Total score *β* (95%CI)	DS: Total score *β* (95%CI)	DS: Physical domain *β* (95%CI)	DS: Emotional domain *β* (95%CI)	DS: Social domain *β* (95%CI)	DS: School domain *β* (95%CI)	DS: Psycho-social domain *β* (95%CI)
Associated disease features	Behavioural problems^a^	−0.6 (−1.0–−0.2)	−1.1 (−1.4–−0.8)	−1.0 (−1.5–−0.5)	−0.9 (−1.2–−0.5)	−1.6 (−2.1–−1.2)	−1.0 (−1.6–−0.5)	−1.2 (−1.5–−0.9)
	Independent walking 500m^b^		8.5 (4.2 −12.8)	18.6 (10.9–26.4)				
	Level of ID—no to borderline ID^c^ 88				−19.8 (−31.4−8.1)	−18.9 (−32.8−5.0)		−10.6 (−20.2–1.1)
	Level of ID—mild ID^c^					−12.6 (−20.6−4.6)		−7.1 (−12.7–1.6)
	Sleeping difficulties			2.4 (0.4–2.1)	−2.9 (−4.2–1.7)			
	GI and eating problems			−4.3 (−6.5–2.1)				
	Autonomic dysfunction		−2.1 (−3.2–1.0)	−3.2 (−5.0–1.3)			−2.8 (−4.7–0.8)	−1.4 (−2.6–0.2)-
	Impact score				−2.6 (−5.1–0.0)			−2.3 (−4.7–0.1)
	Age	−0.3 (−0.5–0.0)		−0.5 (−0.8–0.2)	−0.3 (−0.6–0.1)		0.4 (0.0–0.8)	
	*Adjusted R^2^*	*0.33*	*0.51*	*0.49*	*0.41*	*0.45*	*0.28*	*0.53*

Only disease features that were significantly associated in one or more of the multivariable models are shown.

DS = Dravet syndrome, non-DS = non-Dravet syndrome phenotypes, HRQoL = health-related quality of life, ID = intellectual disability, and GI = gastrointestinal.

*β* = standardized regression beta-coefficients depicted with 95% confidence intervals (95%CI). Adjusted *R*^2^ = the proportion of variance in the outcome measure, explained by the model.

^a^Behavioural problems as measured by the A/CBCL mean *T*-score of the total problem scale.

^b^Compared to use of a wheelchair.

^c^Severe ID level was used as reference for all categories. The category ‘Moderate ID’ was not significantly associated with any of the models and is therefore not included in the table.

Disease characteristics have been reported and compared between the four age groups (Table 3) in the 2022 cross-sectional cohort. Level of ID, use of more than three anti-seizure medication (ASM), the use of contra-indicated medication (CIM), A/CBCL scores and the composite impact score significantly differed between age groups. The use of a wheelchair versus independent walking on the Functional Mobility Scale for 500 m significantly differs when merging the younger age groups and subsequently comparing participants younger than twenty years old (*n* = 76) to those older than twenty years old (*n* = 39). When assessing the composite impact score per age group, there is a significantly lower proportion of participants in the age group 13–20 years old that have a composite impact score of 2–3, compared to the other three groups.

**Table 3:** Baseline characteristics per age group in Dravet syndrome

	2–6 yo	7–12 yo	13–20 yo	>20 yo	
*N* (%)	23 (20.0)	21 (18.3)	32 (27.8)	39 (33.9)	
Gender (% female)	11/23 (47.8)	12/21 (57.1)	13/32 (40.6)	21/39 (53.8)	NS
Developmental level *n* (%)^a^					*P* < 0.0001
1: no to borderline ID	5/23 (21.7)	0	2/32 (6.3)	0	
3: mild ID	12/23 (52.2)	10/21 (47.6)	9/32 (28.1)	6/39 (15.4)	
4: moderate ID	4/23 (17.4)	4/21 (19.0)	12/32 (37.5)	8/39 (20.5)	
5: severe ID	2/23 (8.7)	7/21 (33.3)	9/32 (28.1)	25/39 (64.1)	
					
Mean total E-chess score (SD)	13.8 (3.4)	12.8 (3.7)	12.3 (3.3)	13.1 (2.9)	NS
−*Missing*	1	1	0	0	
Major seizure frequency *n*(%)					NS
- No seizures	0	4/21 (19.0)	2/32 (6.3)	3/39 (7.7)	
- Yearly	7/23 (30.4)	4/21 (19.0)	9/32 (28.1)	6/39 (15.4)	
- Monthly	7/23 (30.4)	6/21 (28.6)	10/32 (31.3)	6/39 (15.4)	
- Weekly	6/23 (26.1)	5/21 (23.8)	6/32 (18.8)	22/39 (56.4)	
- Daily	3/23 (13.0)	2/21 (9.5)	5/32 (15.6)	2/39 (5.1)	
					
Use of ≥3 ASM *n* (%)	20/23 (87.0)	14/20 (70)	19/32 (59.4)	33/39 (84.7)	*P* < 0.05
Use of CIM *n* (%)	3/23 (13.0)	6/20 (30.0)	25/32 (78.1)	38/39 (97.4)	*P* < 0.0001
					
ABCL/CBCL total score *t*-score^b^	65.6 (10.1)	66.2 (7.1)	61.9 (6.2)	58.1 (5.9)	*P* < 0.001
Per category *n*(%):					
- Normal range	8/23 (34.8)	2/19 (10.5)	9/28 (32.1)	18/34 (52.9)	*P* < 0.001
- Borderline	1/23 (4.3)	4/19 (21.1)	6/28 (21.4)	11/34 (32.4)	
- Clinical	14/23 (60.9)	13/19 (68.4)	13/28 (46.4)	5/34 (14.7)	
					
Functional Mobility Scale *n* (%)^c^					NS
- Independent on all surfaces	3/22 (15.0)	7/21 (33.3)	10/30 (33.3)	9/37 (24.3)	
- Independent on flat surfaces	8/22 (40.0)	6/21 (28.6)	11/30 (36.7)	6/37 (16.2)	
- Uses a wheelchair	9/22 (45.0)	8/21 (38.1)	9/30 (30.0)	22/37 (59.5)	
					
Dysautonomia *n* (%):					NS
3 or more symptoms on a weekly basis	11/23 (47.8)	9/21 (42.9)	5/31 (16.1)	12/38 (31.6)	
Gastrointestinal and eating problems: *n* (%)					
3 or more symptoms on a weekly basis	10/23 (43.5)	8/21 (38.1)	7/32 (21.9)	16/39 (41.0)	NS
Sleeping problems, SPSQ^d^ *n* (%)					
Composite Sleep Index > 4	5/23 (21.7)	7/21 (33.3)	3/32 (9.4)	6/39 (15.4)	NS
Composite impact score *n* (%):					*P* < 0.05
- Impact on 0–1 aspect	4/23 (17.4)	2/20 (10.0)	13/30 (43.3)	9/39 (23.1)	
- Impact on 2–3 aspects	19/23 (82.6)	18/20 (90.0)	17/30 (56.7)	30/39 (76.9)	

Prevalence is depicted as occurrence/total (percentage). For continuous variables, missings are displayed separately.

Differences between age groups were tested per characteristic, significance is indicated in the right column.

ASM = anti-seizure medication, CIM = contra-indicated medication, CSI = composite sleeping index as measured by the SPSQ, and NS = not significant SD = standard deviation, yo = years old.

^a^No intellectual disability (ID) = intelligence quotient (IQ) > 85, borderline ID = IQ 70–85, mild ID = IQ 50–70, moderate ID = IQ 30–50, severe to profound ID = IQ < 30.

^b^Based on the Dutch parent report version of the Child Behaviour Checklist 1.5–5 years (CBCL), Child Behaviour Checklist 6–18 years, and the Adult Behaviour Checklist 18–59 years (ABCL).

^c^The outcome for 500-m distance on the Dutch version of the Functional Mobility Scale (FMS).

^d^Based on the Dutch translation of the Sleep Behaviour Questionnaire by Simonds & Parraga (SQ-SP), modified version for use in individuals with intellectual disability.

**Figure 2:**
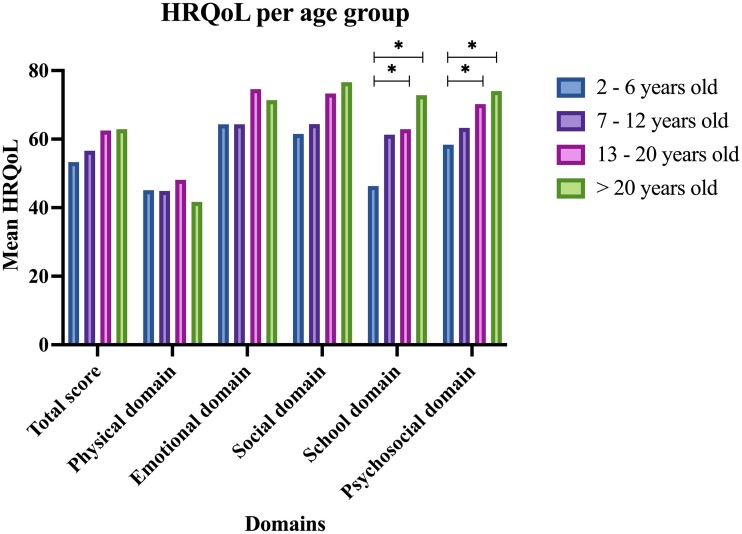
**HRQoL total and domain scores per age group in DS.** Differences between age groups were tested with an ANOVA and post-hoc Tukey HSD. *statistically significant at the *P* < 0.05 level: in the School domain age group 2–6yo versus age group 13–20yo (*F*-value 6.02, *P* < 0.05) and age group 2–6 year old versus age group >20yo (*F*-value 6.02, *P* < 0.001). In the Psychosocial domain age group 2–6yo versus age group 13–20yo (*F*-value 5.1, *P* < 0.05) and age group 2–6yo versus age group >20 year old (F-value 5.09, *P* < 0.01). DS = Dravet syndrome HRQoL = health-related quality of life yo = years old. Figure created with Graphpad Prism.

Figure 2 illustrates the total HRQoL score and the domain scores per age group. The Physical domain score is consistently low across age groups. The oldest two age groups have a significantly higher School and Psychosocial domain score, compared to the youngest age group. Results from the multivariable regression analyses per age group with HRQoL total score as the outcome measure are shown in Table 4. A higher number of signs of autonomic dysfunction was associated with lower HRQoL in all age groups, except the oldest age group. More severe behavioural problems were associated with lower HRQoL for the age groups 2–6 years old, 13–20 years old and >20 years old. In the age group 7–12 years old, a higher total E-chess score, i.e. a higher burden of epilepsy, contributes to lower total HRQoL.

**Table 4:** Results from the multivariable regression analyses for the cross-sectional DS cohort per age group with HRQoL total score as the outcome measure

		HRQoL per age group			
		2–6 yo *β* (95%CI)	7–12 yo *β* (95%CI)	13–20 yo *β* (95%CI)	> 20 yo *β* (95%CI)
Associated disease features	Behavioural problems^a^	−1.2 (−1.7–−0.8)		−1.0 (−1.7–−0.4)	−1.3 (−1.9–−0.3)
	Level of ID—No to mild ID^b^				−13.4 (−24.4–−2.5)
	Total E-Chess score		−1.4 (−2.7–−0.0)		
	GI and eating problems				−5.1 (−7.7–−2.7)
	Autonomic dysfunction	−2.6 (−4.5–−0.6)	−3.9 (−5.9–−1.8)	−3.8 (−6.8–−0.7)	
	*Adjusted R^2^*	*0.61*	*0.51*	*0.47*	*0.40*

Only disease features that were significantly associated with one or more of the multivariable models are shown.

HRQoL = health-related quality of life, ID = intellectual disability, and GI = gastrointestinal yo = years old.

*β* = standardized regression beta-coefficients depicted with 95% confidence intervals (95%CI). Adjusted *R*^2^ = the proportion of variance in the outcome measure, explained by the model.

^a^Behavioural problems as measured by the A/CBCL mean *T*-score of the total problem scale.

^b^Severe ID level was used as reference.

**Figure 3:**
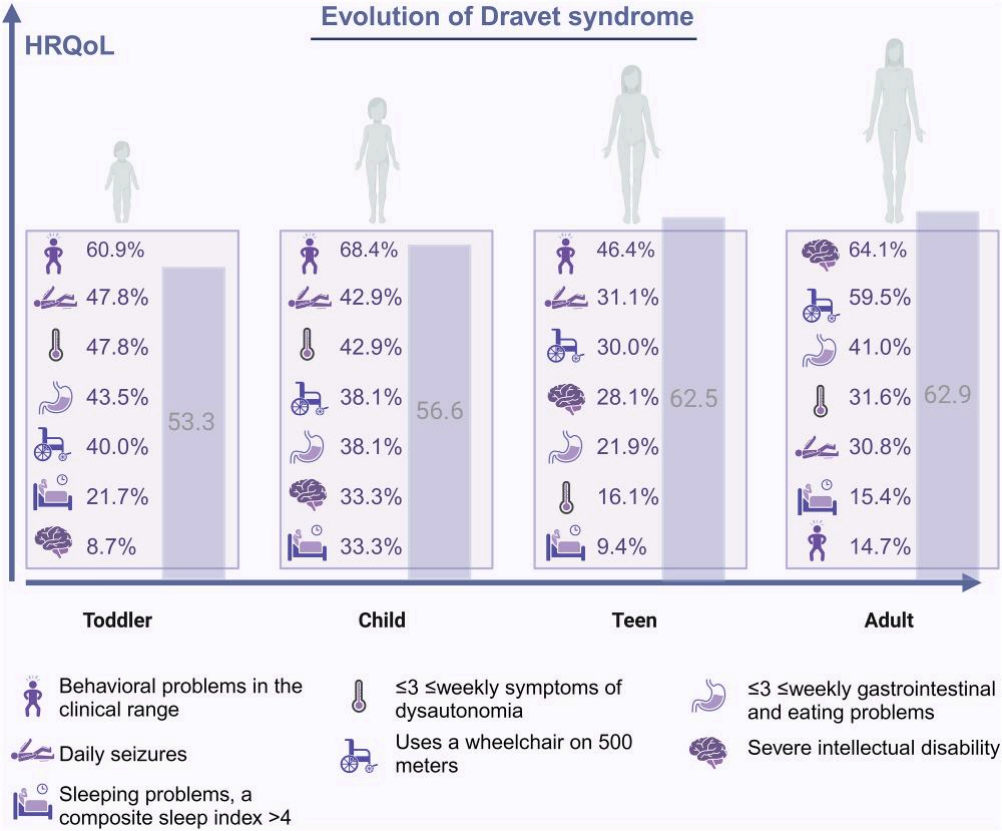
**The evolution of Dravet syndrome.** The prevalence of disease features per age group is depicted in percentages with the icons. The purple bars indicate the total HRQoL score per age group. Behavioural problems are measured with the total problem scale of the Dutch parent-report version of the Child Behaviour Checklist 1.5–5 years (CBCL), Child Behaviour Checklist 6–18 years, and the Adult Behaviour Checklist 18–59 years (ABCL). Sleeping problems are measured with the composite sleep index (>4 indicates a severe sleeping problem), based on the Dutch translation of the Sleep Behaviour Questionnaire by Simonds & Parraga (SQ-SP), modified version for use in individuals with intellectual disability. HRQoL = health-related quality of life. Figure created with Biorender.

Figure 3 graphically illustrates the evolution of disease characteristics and HRQoL in DS across age groups.

### HRQoL in the longitudinal cohort

**Figure 4:**
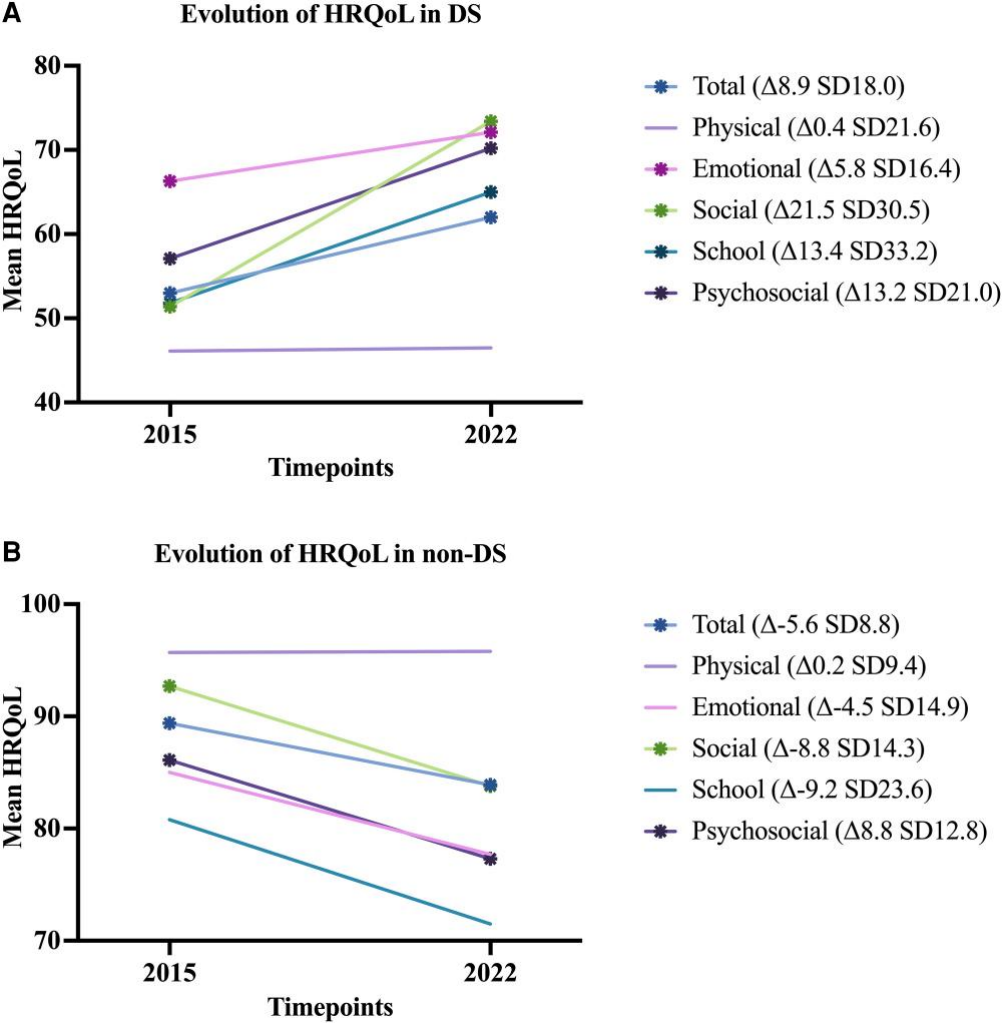
**Change in HRQoL total and domain** scores between the two timepoints, depicted for DS (4A) and non-DS (4B) separately. Addition of an asterisk to the line indicates a significant difference between timepoints (tested with a paired t-test or Wilcoxon signed-rank test), in DS for the total HRQoL score (t-value 3.6, P < 0.001), for the Emotional domain score (t-value 2.6, P < 0.05), for the Social domain score (t-value 5.0, P < 0.0001), for the School domain score (t-value 2.7, P < 0.05) and for the Psychosocial domain score (t-value 4.5, P < 0.0001). In non-DS for the total HRQoL score (t-value −2.3, P < 0.05), for the Social domain score (t-value −2.3, P < 0.05) and for the Psychosocial domain score (t-value −2.5, P < 0.05). DS = Dravet syndrome non-DS = non-Dravet phenotypes (GEFS+/FS) HRQoL = health-related quality of life, as measured with the PedsQl SD = standard deviation.

Sixty-five participants, of whom 52 (80.0%) with DS and 13 (20.0%) with non-DS, completed the PedsQl proxy-reported questionnaire at both timepoints (Supplementary Table 2). The mean age of the DS group was 18.9 years old, compared to 15.3 years old in the non-DS group. The changes in mean scores of disease features between the two timepoints are listed in Supplementary Table 3. Figure 4 represents the mean change in total and domain HRQoL scores between the two timepoints for both DS (Fig. 4A) and non-DS (Fig. 4B). In the DS group, all total and domain scores were significantly higher at the second time point, except the Physical domain score. In the non-DS group, the total score, the Social domain score and Psychosocial domain score were significantly lower.

Table 5 depicts the results from the multivariable regression analysis for DS participants. Less behavioural problems in 2022 as compared to 2015, lower major seizure frequency in 2022 and a higher age were associated with a higher percentage increase in HRQoL score between timepoints, with modest effect sizes. The sample size of the non-DS cohort (*n* = 13) was insufficient for a regression analysis.

**Table 5:** Longitudinal DS cohort with the percentage change in HRQoL from 2015 to 2022 as the outcome measure

		Percentage change in HRQoL *β* (95%CI)
Associated disease features	Percentage change in behavioural problems^a^	−1.2 (−2.0–−0.4)
	Change in major seizure frequency	−0.1 (−0.2–−0.0)
	Age	0.03 (0.01–0.04)
	*Adjusted R^2^*	*0.44*

Only disease features that were significantly associated with the multivariable model are shown.

HRQoL = health-related quality of life.

*β* = standardized regression beta-coefficients depicted with 95% confidence intervals (95%CI). Adjusted *R*^2^ = the proportion of variance in the outcome measure, explained by the model.

^a^Behavioural problems as measured by the percentage change in A/CBCL mean *T*-score of the total problem scale.

Δ = Mean difference between timepoints.

### Interview data

Interview data from the 2022 cross-sectional cohort are depicted in Supplementary Table 4. A negative impact of the disease on family life was reported in 79.5% of DS participants and 42.9% of non-DS, a negative impact on brothers and sisters in 64.3% of DS and 33% of non-DS and a negative impact on the relationship of parents in 64.4% of DS and 14.3% in non-DS. Parents/caregivers listed SUDEP (29.0%), other complications of epilepsy such as status epilepticus and injury during a seizure (24.0%), and worries about the future (20.0%) as their biggest fears. Non-DS participants indicated that their biggest fear was complications of epilepsy (45.5%), followed by others (27.3%), which included shame after a seizure and losing their driver's licence. Parents talked freely about their lived experience and fears. Illustrative quotes are depicted in Supplementary Table 5.

## Discussion

This study provides an overview of the evolution of HRQoL across the lifespan, by performing a cross-sectional and longitudinal assessment of a cohort of *SCN1A*-related seizure disorders. For DS, HRQoL total and domain scores were significantly lower than in the non-DS groups and normative controls. The most compelling finding was a higher HRQoL score with older age in DS, reflected in both our cross-sectional analysis per age group and the longitudinal analysis comparing scores obtained in 2022 to scores obtained in 2015. For non-DS, HRQoL was comparable to the general population in the cross-sectional analysis, but appeared lower than in 2015 in the modest longitudinal non-DS cohort.

When assessing HRQoL scores per age group, the oldest age groups had higher School and Psychosocial domain scores, compared to the youngest age group. All HRQoL domain scores, except the Physical domain score, were significantly higher seven years later. The Physical domain score remained consistently low, which can be attributed to the relatively high mean age of the DS participants (18.2 years in 2022). Gait disturbances are present in 80–90% of DS patients over 10 years old,^10^ which suggests that in our cohort many may have already reached a permanent level of functional motor deficit. This is corroborated by the significantly higher percentage of DS participants older than 20 years that use a wheelchair at 500 m distance, compared to those younger than 20 years old. The higher scores of total HRQoL at older age may be multifactorial. First, standard care for DS has improved importantly over the past years, resulting in a more effective seizure reduction and lower prevalence of comorbidities,^35^ which may be reflected in higher HRQoL scores in 2022. Moreover, as Dravet participants age, there is often a decrease in epilepsy severity and a ceiling of neurodevelopment.^4,10,36^ In our cohort and in previous work,^16^ the prevalence of severe behavioural problems declined when reaching adulthood. Possibly, reaching a phase of relative disease stability, i.e. the decrease in seizure burden and behavioural problems in combination with the plateauing of other disease features, may have a positive effect on the well-being of both DS patients and their parents. Lastly, HRQoL is based on a proxy-reported questionnaire, completed by parents. Therefore, the resilience and well-being of parents are likely to influence the results. The negative psychosocial implications of DS on parents/caregivers and siblings have been well described.^37-39^ Parents may have developed coping mechanisms over time to ameliorate the burden of care.^40^ The ceiling effect of disease manifestations likely explains why our findings differ from the sole previous longitudinal study on HRQoL in DS, with a 10-year follow-up of 68 patients, who reported a deterioration of HRQoL at the second timepoint, which was—however—only significant in the younger age group, 0–5 years old at baseline.^14^ On average, our cohort was older, so it is likely the ceiling effect was more often seen. This may imply that these results are not generalizable to younger DS patients.

Behavioural problems were most consistently associated with HRQoL, both in the cross-sectional and in the longitudinal cohort. Behavioural problems are highly prevalent in DS,^10,13,22^ and have previously been shown to significantly diminish HRQoL.^13,14,41^ We recently published a characterization of the behaviour profile of this cohort, in which parents of 82.5% of DS participants reported the presence of behavioural difficulties.^22^ On the total problems scale of the A/CBCL questionnaires, 43% of DS participants scored in the clinical (severe) range. Associated factors were younger age and sleeping difficulties. The syndrome subscales ‘attention problems’ and ‘aggressive behaviour’ were implicated most, with respectively 37.4% and 15.0% of DS participants scoring in the clinical range. In 34.1% of participants, parents reported that there had not been any form of treatment or guidance, suggesting that behavioural problems may be underreported or underrecognized in clinical practice.^22^ These findings further underline the need for appropriate attention and care for behavioural problems in DS. Transforming standard care into a multidisciplinary approach that includes a psychiatrist or psychologist may be the first step in early recognition and treatment. Different types of behavioural problems require different types of management, either with therapy or medicinally, and should include guidance of both patient and parent. Addressing sleeping problems, for instance, by prescribing melatonin (when indicated), sleep counselling, and assessing the effect and timing of ASM administration, may also have a positive effect on behavioural problems.^42,43^

Earlier studies highlighted the impact of seizure severity on HRQoL in DS.^14,16,17,41^ We observed this association in the longitudinal cohort and in the age group 7–12 years old. These associations correspond to the natural course of epilepsy in DS. Between 1 and 5 years of age, epilepsy worsens with respect to seizure types, frequency and duration, after which it stabilizes and subsequently starts to subside from 8 years and older.^36^ The age group 7–12 years old is therefore a pivotal period with regard to the impact of seizures on daily life. Autonomic dysfunction, a relatively unexplored disease feature, impacted HRQoL prominently in our cross-sectional cohort. Over the past years, several caregiver survey studies reported a high prevalence of signs of dysautonomia, in 50–80% of patients with DS.^8-10^ As a direct effect, symptoms of autonomic dysfunction such as temperature dysregulation, orthostasis, and diaphoresis might cause great discomfort, resulting in lower HRQoL. Indirectly, the presence of autonomic dysfunction might reflect the severity of the disease.^44^ The higher the number of symptoms of autonomic dysfunction, the more severe the disease phenotype. The use of a wheelchair on 500 m was associated with lower HRQoL, which corresponds to previous studies.^18^ A limited mobility poses a high burden on daily life and coincides with a higher risk of concomitant disease.

For the non-DS group, HRQoL was comparable to a healthy control population, for both the self-report and the proxy-reported group. This unexpected finding may be due to the heterogeneity of the non-DS group, which also included seizure-free participants with a history of FS. The total HRQoL score and the Social and Psychosocial domain scores were significantly lower compared to 2015 in the proxy-reported group. Possibly, growing up with epilepsy is known to affect HRQoL in general^45^ and the stigma and impact on social life likely affect HRQoL in this group as well. However, the sample size (*n* = 13) was too small for robust conclusions.

In the telephonic interviews, parents expressed the impact the disease has on their daily lives. In the Dravet cohort, the association between a higher impact score and lower Emotional and Psychosocial domain scores reflects the interaction between the parents’ experiences and the patient's psychological well-being. Surprisingly, the proportion of participants in the age group 13–20 years old that scored on 2–3 aspects of the composite impact score was lower than in the other three groups. Perhaps, the impact on parents and siblings is higher in the younger age groups since this is a more tumultuous phase of disease, and siblings are also younger, compared to the age group 13–20 years old. The high impact scores in the oldest age group may reflect the concerns of both parents and siblings about the responsibilities involved in taking care of their child/sibling with Dravet syndrome in the future. The quotes from the telephonic interview, included in the Supplementary material, illustrate the constant stress and anxiety caregivers experience, with fear of SUDEP and the uncertain future of their child. These findings further emphasize the importance and the need for tools to increase empowerment.

There are several limitations to this study. For several disease characteristics, non-validated questionnaires were used. Validated questionnaires for autonomic dysfunction are either very extensive, burdensome for the participant/caregiver, or based on data from physical examination which were unavailable to us. No validated questionnaires on gastrointestinal and eating problems were applied to our study population. Since these topics were marked by parent focus groups^9,11^ as highly relevant, we assessed these disease features with self-devised questionnaires. Moreover, we used the PedsQl to assess HRQoL. Other measures may have been more sensitive for individuals with ID (QI-disability). However, in order to perform a longitudinal analysis, we selected the measure chosen for the 2015 studies.^13,18^ Third, all questionnaires in DS patients were proxy-reported. Inevitably, the measures fully based on questionnaires will have been influenced by the parent's well-being. To partly account for parental distress, we included the composite impact score in our analyses. Fourthly, the cross-sectional data for the total DS cohort was right-skewed in age distribution. However, by stratifying analyses based on age, we could segregate the influence on the results per age group. Fifthly, there were some significant differences between the responders and non-responders from the 2015 cohort. However, since the cross-sectional analysis per age group and the longitudinal analysis showed similar results, this is not likely to have influenced the conclusion of the study. Lastly, recent work has delineated an early onset *SCN1A-*phenotype, caused by a gain-of-function mutation,^46^ for which we did not screen our cohort. However, since the gain-of-function (GOF) phenotype is very rare, with a distinguished clinical phenotype, we do not expect to have wrongly diagnosed GOF phenotypes as Dravet and thus do not expect an impact on our results.

This study has provided a comprehensive overview of the evolution of HRQoL and other disease features in *SCN1A-*related seizure disorders. Based on the age-specific cross-sectional data, we detailed the natural course of DS in Fig. 3, which could function as a blueprint for treating physicians to provide relevant age-specific care. Seven years after the first assessment, HRQoL was significantly higher in the DS participants. This improvement is likely multifactorial, reflecting the fruits of the current advanced care strategy, the ceiling of severity of several disease symptoms and possibly an enhanced level of social and emotional wellbeing of parents and patients. The evident impact of behavioural problems on HRQoL across age groups and in the long term stresses the importance of incorporating multidisciplinary, age-specific management into standard care. Assessment of relatively unexplored disease features, such as autonomic dysfunction that affect HRQoL in specific age groups and domains, will enable tailored care, to improve HRQoL.

## Supplementary Material

fcae285_Supplementary_Data

## Data Availability

The data that support the findings of this study are available on request from the corresponding author. The data are not publicly available due to privacy or ethical restrictions. The codes used are available via a public repository on Github, which can be accessed through this link: https://github.com/CristaMinderhoud/HRQoL-in-SCN1A.git.
