# Supplementary material for: Quality of life in SCN1A-related seizure disorders across the lifespan
Source: Brain Commun. 2024 Aug 26;6(5):fcae285. doi: 10.1093/braincomms/fcae285 (PMC11375853; doi:10.1093/braincomms/fcae285)
Supplement: fcae285_Supplementary_Data [file fcae285_supplementary_data.docx]

# **Supplementary material**

Supplementary Figure 1. Flowchart illustrating inclusion and data collection at both timepoints.


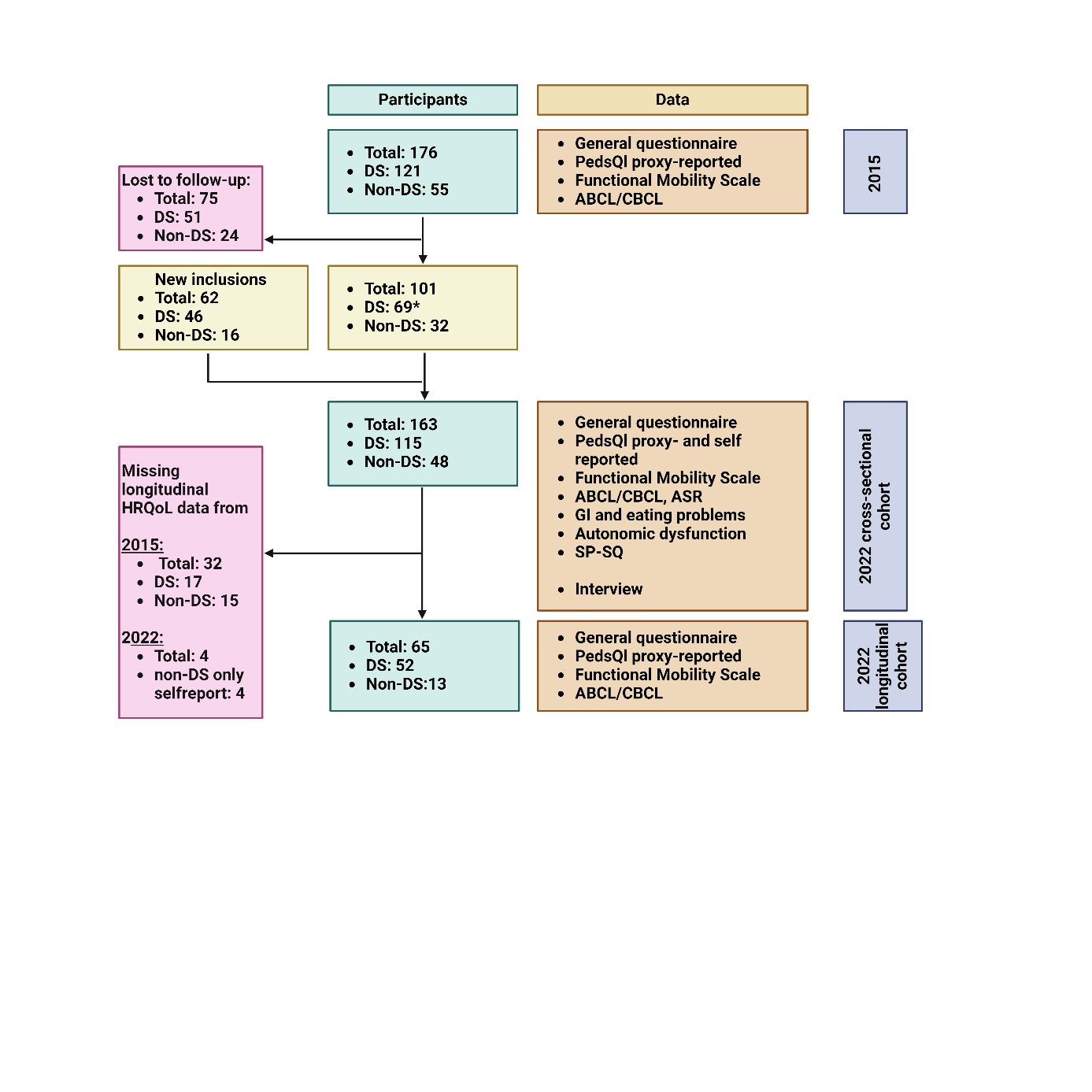
*1 patient was classified as Dravet syndrome in 2015 and was reclassified as GEFS+ in 2022.
DS=Dravet syndrome non-DS=non-Dravet syndrome PedsQl= Pediatric Quality of Life Inventory ABCL= Adult Behavior Checklist 18-59 years CBCL=Child Behavior Checklist 1.5-5years and 6-18 years ASR=Adult Self-report >18 years GI= gastrointestinal SP-SQ= Dutch translation of the Sleep Behavior Questionnaire by Simonds & Parraga (SQ-SP), modified version for use in individuals with intellectual disability

Supplementary questionnaire 1: Questionnaire gastrointestinal and eating problems
(original questionnaire is in Dutch)

Cross out which options are not applicable

1. Does your child experience the following symptoms and if so, how often?
   1. Problems with chewing?

Never or almost never / monthly / weekly / daily / every meal / in phases, so frequency differs

- 1. Problems with swallowing?
     Never or almost never / monthly / weekly / daily / every meal / in phases, so frequency differs
  2. Throwing up?
     Never or almost never / monthly / weekly / daily / every meal / in phases, so frequency differs
  3. Loss of appetite?

Never or almost never / monthly / weekly / daily / every meal / in phases, so frequency differs

Constipation?

Never or almost never / monthly / weekly / daily / every meal / in phases, so frequency differs

- 1. Easily distracted during mealtimes?

Never or almost never / monthly / weekly / daily / every meal / in phases, so frequency differs

- 1. Temper tantrums during mealtimes?

Never or almost never / monthly / weekly / daily / every meal / in phases, so frequency differs

1. Is your child a picky eater?
   1. Yes
   2. No
2. Is your child currently experiencing any issues with eating or drinking?
   1. Yes
   2. No
3. Did your child experience difficulties with eating or drinking in the first year of life?
   1. Yes
   2. No
4. Does your child have a PEG-tube or nasogastric tube?
   1. Yes, for partly feeding
   2. Yes, for full feeding
   3. No
5. If you child has a PEG-tube or nasogastric tube, has the daily life of both you and your child improved since the use of a PEG-tube or nasogastric tube?
   1. Yes
   2. No
6. If there are eating problems, did they start because of the ketogenic diet?
   1. Yes
   2. No
7. Do the eating problems greatly impact your daily life?
   1. Yes
   2. No

Length of your child:

Weight of your child:

Supplementary questionnaire 2: questionnaire on autonomic dysfunction (original questionnaire was in Dutch)

Do the following symptoms occur (in your child) for a duration of at least ten minutes, outside of an epileptic seizure and without an environmental trigger, and how often?

Cross out which options are not applicable.

1. Cold hands or feet?

Never or hardly ever / monthly / weekly / several times per week / daily

1. One side of the body warmer or colder than the other?

Never or hardly ever / monthly / weekly / several times per week / daily

1. Fiery red or blueish discoloration of hands or feet?

Never or hardly ever / monthly / weekly / several times per week / daily

1. Excessive sweating?

Never or hardly ever / monthly / weekly / several times per week / daily

1. A decreased or inability to sweat?

Never or hardly ever / monthly / weekly / several times per week / daily

1. Dilated pupils?

Never or hardly ever / monthly / weekly / several times per week / daily

1. Slow emptying of the stomach?

Never or hardly ever / monthly / weekly / several times per week / daily

1. Episodes of tachycardia not induced by exercise, excitement or other identifiable cause?

Never or hardly ever / monthly / weekly / several times per week / daily

1. Episodes of blushing of the face or chest not induced by exercise, excitement or other identifiable cause?

Never or hardly ever / monthly / weekly / several times per week / daily

1. Tingling of hands or feet?

Never or hardly ever / monthly / weekly / several times per week / daily

1. Signs of orthostasis (dizzy when standing up)?

Never or hardly ever / monthly / weekly / several times per week / daily

1. Occurrence of at least 4 symptoms simultaneously

Never or hardly ever / monthly / weekly / several times per week / daily

Supplementary table 1: descriptive data responders versus non-responders (2015 data)

1. Dravet participants

|  | Non-responders | Responders |  |
| --- | --- | --- | --- |
| N | 51 | 70 |  |
| Death n (%) | 8 (15.7) | 0 |  |
| Mean age (SD) | 19.5 (11.8) | 14.1 (9.0) | **P<0.05** |
|  |  |  |  |
| Developmental level n (%)^a^ |  |  |  |
| 1: no ID | 3 (5.9) | 2 (2.9) | NS |
| 2: borderline ID | 5 (9.8) | 6 (8.6) |  |
| 3: mild ID | 5 (9.8) | 17 (24.3) |  |
| 4: moderate ID | 10 (19.6) | 21 (30.0) |  |
| 5: severe ID | 28 (54.9) | 24 (34.3) |  |
|  |  |  |  |
| Mean age at seizure onset in months (SD) | 6.2 (4.5) | 5.8 (3.0) | NS |
|  |  |  |  |
| Major seizure frequency n (%) |  |  | NS |
| - No major seizures | 3 (5.9) | 5 (7.1) |  |
| - Yearly | 6 (11.8) | 10 (14.3) |  |
| - Monthly | 11 (21.6) | 18 (25.7) |  |
| - Weekly | 23 (45.1) | 29 (41.4) |  |
| - Daily | 8 (15.7) | 8 (11.4) |  |
|  |  |  |  |
| ABCL/CBCL total score mean t-score^b^ | 59.2 (9.8) | 63.1 (6.9) | NS |
|  |  |  |  |
| Functional Mobility Scale  n (%)^c^ |  |  | **P<0.05** |
| - Independent on all surfaces | 2 (10.0) | 16 (36.4) |  |
| - Independent on flat surfaces | 5 (25.0) | 14 (31.8) |  |
| - Uses a wheelchair | 13 (65.0) | 14 (31.8) |  |
| - *Missing* | 31 | 26 |  |
|  |  |  |  |
| HRQoL |  |  |  |
| - Mean total score (SD) | 54.5 (19.0) | 53.4 (15.6) | NS |

1. Non-DS participants

|  | Non-responders | Responders |  |
| --- | --- | --- | --- |
| N | 24 | 31 |  |
| Death n | 0 | 0 |  |
| Mean age (SD) | 32.5 (21.4) | 23.6 (19.1) | NS |
|  |  |  |  |
| Developmental level n (%)^a^ |  |  | NS |
| 1: no ID | 21 (87.5) | 29 (93.5) |  |
| 2: borderline ID | 3 (12.5) | 2 (6.5) |  |
| 3: mild ID | 0 | 0 |  |
| 4: moderate ID | 0 | 0 |  |
| 5: severe ID | 0 | 0 |  |
|  |  |  |  |
| Mean age at seizure onset in months (SD) | 14.9 (8.7) | 14.5 (13.7) | NS |
|  |  |  |  |
| Major seizure frequency n (%) |  |  | NS |
| - No major seizures | 19 (79.2) | 21 (67.7) |  |
| - Yearly | 4 (16.7) | 9 (29.0) |  |
| - Monthly | 1 (4.2) | 1 (3.2) |  |
| - Weekly | 0 | 0 |  |
| - Daily | 0 | 0 |  |
|  |  |  |  |
| ABCL/CBCL total score mean t-score^b^ | 55.8 (10.8) | 48.5 (10.1) | **P<0.05** |
|  |  |  |  |
| Functional Mobility Scale  n (%)^c^ |  |  | NS |
| - Independent on all surfaces | 6 (100.0) | 14 (100.0) |  |
| - Independent on flat surfaces | 0 | 0 |  |
| - *Missing* | 18 | 17 |  |
|  |  |  |  |
| HRQoL^d^ |  |  |  |
| - Mean total score (SD) | 86.7 (11.9) | 90.8 (5.3) | NS |

Note: 1 patient was classified as Dravet syndrome in 2015 and was reclassified as GEFS+ in 2022.

ID=intellectual disability HRQoL=health-related quality of life SD=standard deviation ^a^no intellectual disability (ID) = intelligence quotient (IQ) 85, borderline ID = IQ 70-85, mild ID= IQ 50-70, moderate ID = IQ 30-50, severe to profound ID= IQ <30

^b^ Based on the Dutch parent report version of the Child Behavior Checklist 1.5–5 years (CBCL), Child Behavior Checklist 6–18 years, the Adult Behavior Checklist 18–59 years (ABCL)
^c^ The outcome for 500 meters distance on the Dutch version of the Functional Mobility Scale (FMS)

^d^Based on the Pediatric Quality of Life Inventory (PedsQl).

Supplementary table 2. Baseline characteristics of the longitudinal cohort assessed in 2022.

|  | Dravet | Non-DS proxy-reported |
| --- | --- | --- |
| N (%) | 52 (80.0) | 13 (20.0) |
| Mean age (SD) | 18.2 (6.8) | 12.6 (2.9) |
| Gender (% female) | 22 (42.3) | 11 (84.6) |
|  |  |  |
| Developmental level n (%)^a^ |  |  |
| 1: no ID | 0 | 13 (100.0) |
| 2: borderline ID | 1 (1.9) | 0 |
| 3: mild ID | 12 (23.1) | 0 |
| 4: moderate ID | 16 (30.8) | 0 |
| 5: severe ID | 23 (44.2) | 0 |
| - *Missing* | 0 | 0 |
|  |  |  |
| Mean age at seizure onset in months (SD) | 5.9 (3.1) | 9.2 (3.2) |
| - *Missing* | 0 | 0 |
|  |  |  |
| Mean total E-chess score | 12.4 (2.9) | 6.6 (2.3) |
| - *Missing* | 1 | 1 |
| Daily seizures n (%) | 16 (30.8) | 0 |
| - *Missing* | 0 | 0 |
|  |  |  |
| Major seizure frequency n (%) |  |  |
| - No major seizures | 5 (9.6) | 6 (46.2) |
| - Yearly | 9 (17.3) | 6 (46.2) |
| - Monthly | 16 (30.8) | 1 (7.7) |
| - Weekly | 15 (28.8) | 0 |
| - Daily | 7 (13.5) | 0 |
| - *Missing* | 0 | 0 |
|  |  |  |
| ABCL/CBCL total score mean t-score^b^ | 61.9 (6.9) | 54.7 (7.5) |
| - *Missing* | 5 | 2 |
|  |  |  |
| Functional Mobility Scale  n (%)^c^ |  |  |
| - Independent on all surfaces | 16 (32.7) | 11 (100.0) |
| - Independent on flat surfaces | 14 (28.6) | 0 |
| - Uses a wheelchair | 19 (38.8) | 0 |
| - *Missing* | 3 | 2 |
|  |  |  |
| Impact score n(%):   - No impact | 8 (16.3) | 4 (44.4) |
| - Impact on 1 aspect | 3 (6.1) | 2 (22.2) |
| - Impact on 2 aspects | 18 (36.7) | 2 (33.3) |
| - Impact on 3 aspects | 20 (40.8) | 0 |
| - *Missing* | 3 | 5 |
|  |  |  |

ID=intellectual disability SD= standard deviation ^a^no intellectual disability (ID) = intelligence quotient (IQ)>85, borderline ID = IQ 70-85, mild ID= IQ 50-70, moderate ID = IQ 30-50, severe to profound ID= IQ<30

^b^ Based on the Dutch parent report version of the Child Behavior Checklist 1.5–5 years (CBCL), Child Behavior Checklist 6–18 years, the Adult Behavior Checklist 18–59 years (ABCL)
^c^ The outcome for 500 meters distance on the Dutch version of the Functional Mobility Scale (FMS)

^d^ Based on the Dutch translation of the Sleep Behavior Questionnaire by Simonds & Parraga (SQ-SP), modified version for use in individuals with intellectual disability

Supplementary table 3. Mean difference in comorbidities (2022 – 2015)

1. Dravet participants

|  | 2015 | 2022 |  |
| --- | --- | --- | --- |
| Developmental level^a^ n (%) |  |  | p<0.05 |
| - No ID | 2 (3.8) | 0 |  |
| - Borderline ID | 6 (11.5) | 1 (1.9) |  |
| - Mild ID | 14 (26.9) | 12 (23.1) |  |
| - Moderate ID | 18 (34.6) | 16 (30.8) |  |
| - Severe ID | 12 (23.1) | 23 (44.2) |  |
|  |  |  |  |
| FMS-500m^c^ n (%) |  |  | NS |
| - Independent on all surfaces | 15 (38.5) | 16 (32.7) |  |
| - Independent on flat surfaces | 14 (35.9) | 14 (28.6) |  |
| - Use of a wheelchair | 10 (25.6) | 19 (38.8) |  |
|  |  |  |  |
| Epilepsy severity |  |  |  |
| *Total E-chess score (SD) | 13.2 (3.1) | 12.4 (2.9) | NS |
| *Frequency major seizures n (%) |  |  | NS |
| - No seizures | 4 (7.7) | 5 (9.6) |  |
| - Yearly | 10 (19.2) | 9 (17.3) |  |
| - Monthly | 12 (23.1) | 16 (30.8) |  |
| - Weekly | 19 (36.5) | 15 (28.8) |  |
| - Daily | 7 (13.5) | 7 (13.5) |  |
|  |  |  |  |
|  |  |  |  |
| ABCL/CBCL/ASR t-score for total problems ^b^ (SD) | 63.5 (8.1) | 61.9 (6.9) | NS |

1. Non-Dravet participants proxy-reported

|  | 2015 | 2022 |  |
| --- | --- | --- | --- |
| Developmental level^a^ n (%) |  |  |  |
| - No ID | 13 (100.0) | 13 (100.0) | NA |
|  |  |  |  |
|  |  |  |  |
| FMS-500m^c^ n (%) |  |  |  |
| - Independent on flat surfaces | 13 (100.0) | 13 (100.0) | NA |
|  |  |  |  |
| Epilepsy severity |  |  |  |
| *Total E-chess score (SD) | 5.9 (1.7) | 6.6 (2.3) | NS |
| *Frequency major seizures n (%) | -0.1 (1.1) | 0 (0.5) |  |
| - No seizures | 6 (46.2) | 6 (46.2) | NS |
| - Yearly seizures | 6 (46.2) | 6 (46.2) |  |
| - Monthly | 1 (7.7) | 1 (7.7) |  |
|  |  |  |  |
| ABCL/CBCL/ASR t-score for total problems ^b^ (SD) | 44 (10.8) | 54.7 (7.5) | P<0.05 |

ID=intellectual disability SD=standard deviation ^a^no intellectual disability (ID) = intelligence quotient (IQ)>85, borderline ID = IQ 70-85, mild ID= IQ 50-70, moderate ID = IQ 30-50, severe to profound ID= IQ <30

^b^ Based on the Dutch parent report version of the Child Behavior Checklist 1.5–5 years (CBCL), Child Behavior Checklist 6–18 years, the Adult Behavior Checklist 18–59 years (ABCL)
^c^ The outcome for 500 meters distance on the Dutch version of the Functional Mobility Scale (FMS)

Supplementary table 4. Interview data

|  | Dravet | Non-DS proxy-reported | Non-DS self-reported |
| --- | --- | --- | --- |
| Interview with n (%) | 112 | 17 | 27 |
| - mother | 93 (83.0) | 12 (70.6) | 0 |
| - father | 13 (11.6) | 4 (23.5) | 1 (8.0) |
| - self | 0 | 1 (5.9) | 22 (88.0) |
| - both parents | 5 (4.5) | 0 | 0 |
| - sibling | 1 (0.9) | 0 | 0 |
| - partner | 0 | 0 | 2 (8.0) |
| - *Missing* | 3 | 4 | 2 |
|  |  |  |  |
| Impact on family n (%) |  |  |  |
| - In the past | 13 (11.6) | 0 | 9 (36.0) |
| - Currently | 89 (79.5) | 9 (52.9) | 7 (28.0) |
| - *Missing* | 3 | 4 | 2 |
|  |  |  |  |
| Impact on brothers/sisters n (%) |  |  |  |
| - N/A | 16 (14.3) | 1 (6.3) | 6 (26.1) |
| - Yes | 72 (64.3) | 6 (37.5) | 7 (30.4) |
| - *Missing* | 3 | 5 | 4 |
|  |  |  |  |
| Impact on relationship of parents n (%) |  |  |  |
| - Only positive | 9 (8.7) | 1 (7.7) | 1 (12.5) |
| - Negative | 67 (64.4) | 2 (15.4) | 1 (12.5) |
| - *Missing* | 10 | 8 | 19 |
|  |  |  |  |
| Composite impact score n (%) |  |  |  |
| - No impact | 12 (10.7) | 7 (41.2) | 12 (48.0) |
| - Impact on 1 aspect | 16 (14.3) | 3 (17.6) | 9 (36.0) |
| - Impact on 2 aspect | 40 (35.7) | 7 (41.2) | 4 (16.0) |
| - Impact on 3 aspect | 44 (39.3) | 0 | 0 |
| - *Missing* | 3 | 4 | 2 |
|  |  |  |  |
| Fear of complications n (%) | 100 (89.3) | 13 (76.5) | 7 (28.0) |
| Biggest fear n (%) |  |  |  |
| - SUDEP | 29 (29.0) | 2 (11.8) | 1 (4.0) |
| - Uncertain future | 20 (20.0) | 1 (5.9) | 0 |
| - Psychomotor regression/regression in functioning | 13 (13.0) | 1 (5.9) | 1 (4.0) |
| - Worsening epilepsy | 3 (2.7) | 2 (11.8) | 1 (4.0) |
| - Other complications of epilepsy^a^ | 21 (18.8) | 5 (29.5) | 2 (8.0) |
| - Other | 11 (11.0) | 2 (11.8)) | 4 (16.0) |
| - N/A | 15 (13.4) | 4 (23.5) | 16 (64.0) |
| - *Missing* | 3 | 4 | 2 |
|  |  |  |  |
| Fear of SUDEP n (%) | 71 (63.4) | 6 (35.3) | 3 (12.0) |
| *Missing* | 3 | 4 | 2 |
|  |  |  |  |
| Fear discussed with treating physician n (%) | 50 (50.0) | 4 (23.5) | 3 (12.0) |
| - *Missing* | 4 | 4 | 2 |
|  |  |  |  |

SUDEP=Sudden death in epilepsy
^a^ Such as status epilepticus, worsening of epilepsy, not detecting a seizure, damage during seizure

Supplementary table 5. Quotes from parents of participants with DS from the telephonic interview.

| **Impact family**  *‘Nothing was possible anymore, the seizures completely controlled our lives. As a mother, I felt quilty towards the rest of the kids and others. I lived in total isolation, I was always at home with a sick kid’*  *‘Enormous impact, the whole family is limited in everything, in daily life. You can’t just go out and do things.’*  *‘I have had multiple burn-outs and was in therapy. But now I’m doing much better. He is impacting my life positively now, because I’m learning a lot about myself. I want to help other parents.’*  *‘As parents, we are anxious for the future and where our daughter is going to live. Everything is about her. We built everything, our whole life, around her.’*  *‘The impact was enormous, especially in the first years. Everything was about her. We split up the family with the other kids all the time.’*  *‘My husband is at home with a burn-out. When our daughter lived at home, it became impossible with the many nightly seizures to be able to function at work.’*  *‘The impact was huge, I have felt very guilty for a very long time towards the other kids.’*  *‘The impact is less now, because he doesn’t live at home anymore. Before that we were more caretakers than actual parents.’*  *‘The behavioral problems had a huge impact. Also the constant alertness, always quick to respond when he has an attack, never going on vacation abroad. Always carrying his medication, always scanning the surroundings, checking where the nearest hospital is. He needs structure in everything in his life which is a lot of effort.’*  *‘The disease was all consuming. The impact was not only negative, the disease has been lifechanging in a positive way as well. As a mother, I live my life differently now, everything is relative.’*  *‘There were many things we didn’t do, birthdays, vacations. The family was always split up. As a mother, I quit my job. Our social life is completely different.’* | **Impact other kids**  ‘*Older brother used to stand next to her when she had a seizure and wouldn’t know what to do. Nowadays he feels responsible for her, he is curator with us.’*  *‘It impacted his older sister greatly. Parents can never do something with her together, always just one of them. Daughter worries about parents.’*  *‘Her two older sisters received very little attention from parents when they were younger. The eldest sister couldn’t cope and left the house when she was 16. It really damaged the relationship between mother and daughter.’*  *‘The other kids grew up faster, they had to take care of themselves more often.’*  *‘It highly impacted older brother. He had a lot of anger management issues. It was an intense time, a lot of seizures, and he had to stay with the neighbors or he had to come in the ambulance. He received psychotherapy for this.’*  ‘*The effect on his little sister is big. She starts to panic when the ambulance arrives whilst she is at school.’*  ‘*Huge impact. In the beginning it was all about her. The others could sometimes go on vacation or do fun things but all the fun things always had to stop when she got a seizure.’*  *‘Experienced a lot early in their life, for instance finding their sister blue in bed. It resulted in a lot of behavioral problems in the oldest child. The youngest is now seeking psychotherapy.’*  ‘*All the attention is on his sister (with DS). He is suffering from her behavioral problems, sometimes she hits him in the face.’*  *‘Because the attention was always on their sibling, they’ve grown up to be very flexible and independent.’*  ‘*In the first ten years, the impact was huge. Older sister was psychologically struggling, she often when to school crying. Now that she is a mother herself, she sometimes hears her own child snoring and immediately thinks he is experiencing a seizure, even though he does not have epilepsy. It takes a while to calm down after that. For the other kids also very difficult, a lot of anger and sadness.’* |
| --- | --- |
| **Biggest fear**  *‘That she will not come out of a seizure and will lose her life.’*  *‘The future, he is really happy now, but I fear it will not stay that way.’*  *‘SUDEP’*  *‘The future, I’m afraid that there is no room for her in this world.’*  *‘I’m afraid of what will happen to her if I am old or if I’m no longer here.’*  *‘That he gets a seizure in the night that we miss.’*  *‘Fear of not coming out of a seizure, fear for the future if we are no longer here and he has to live in an institution. Who is going to take care of him?’*  *‘That she will not be able to walk anymore.’*  *‘That she will not be able to do anything anymore and she becomes what they expected her to become.’*  *‘Brain damage or death due to a seizure.’*  *‘Behavioral problems, namely if she gets to puberty.’*  *‘What it will be like when he gets older, caring for him is already difficult because he does not develop anymore. We’re also scared that something else happens to him, for instance when he is with a specialized baby sitter that he will run out into the street.’* | **How often fear of SUDEP**  ‘*Not often, I don’t want this to take over my life.’*  *‘Always in the background.’*  *‘Often, mainly when he is not feeling well.’*  ‘*Regularly, three years ago we had a schooling about it and that was nice.’*  ‘*It used to always be there at times of status epilepticus. During that time always in the back of my mind. Now I don’t really think about it anymore. I have accepted that it is a risk.’*  ‘*In the past continuously. Nowadays rarely.’*  *‘During a period with many seizures or if he stays in the seizure longer, I worry more.’*  ‘*I only heard three years ago that the seizures were deadly, even though she is already an adult, so since then I think about it more.’*  ‘*Every night.’*  *‘I have had trauma treatment (EMDR) because I was constantly thinking about it.’*  ‘*When he is ill, I think about it more.’*  ‘*Every seizure.’* |
